# Cromoglycate drugs suppress eicosanoid generation in U937 cells by promoting the release of Anx-A1

**DOI:** 10.1016/j.bcp.2009.03.010

**Published:** 2009-06-15

**Authors:** Samia Yazid, Egle Solito, Helen Christian, Simon McArthur, Nicolas Goulding, Roderick Flower

**Affiliations:** a Biochemical Pharmacology, William Harvey Research Institute, Bart's and the London School of Medicine and Dentistry, Charterhouse Square, London EC1 M 6BQ, UK; b Department of Cellular and Molecular Neuroscience, Division of Neurosciences and Mental Health, Faculty of Medicine, Imperial College London, Hammersmith Hospital, Burlington Danes Building, Du Cane Road, London W12ONN, UK; c Department of Physiology, Anatomy & Genetics, The University of Oxford, South Parks Road, Oxford OX1 3QX, UK

**Keywords:** Sodium nedocromil, Glucocorticoids, Okadaic acid, PKC, PP2A phosphatase

## Abstract

Using biochemical, epifluorescence and electron microscopic techniques in a U937 model system, we investigated the effect of anti-allergic drugs di-sodium cromoglycate and sodium nedocromil on the trafficking and release of the anti-inflammatory protein Annexin-A1 (Anx-A1) when this was triggered by glucocorticoid (GC) treatment. GCs alone produced a rapid (within 5 min) concentration-dependent activation of PKCα/β (Protein Kinase C; EC 2.7.11.13) and phosphorylation of Anx-A1 on Ser^27^. Both phosphoproteins accumulated at the plasma membrane and Anx-A1 was subsequently externalised thereby inhibiting thromboxane (Tx) B_2_ generation. When administered alone, cromoglycate or nedocromil had little effect on this pathway however, in the presence of a fixed sub-maximal concentration of GCs, increasing amounts of the cromoglycate-like drugs caused a striking concentration-dependent enhancement of Anx-A1 and PKCα/β phosphorylation, membrane recruitment and Anx-A1 release from cells resulting in greatly enhanced inhibition of TxB_2_ generation. GCs also stimulated phosphatase accumulation at the plasma membrane of U937 cells. Both cromoglycate and nedocromil inhibited this enzymatic activity as well as that of a highly purified PP2A phosphatase preparation. We conclude that stimulation by the cromoglycate-like drugs of intracellular Anx-A1 trafficking and release (hence inhibition of eicosanoid release) is secondary to inhibition of a phosphatase PP2A (phosphoprotein phosphatase; EC 3.1.3.16), which probably forms part of a control loop to limit Anx-A1 release. These experiments provide a basis for a novel mechanism of action for the cromolyns, a group of drugs that have long puzzled investigators.

## Graphical abstract

**Figure:**
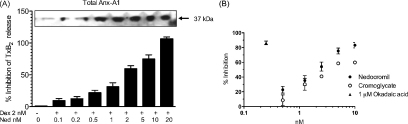

## 1 Introduction

Anx-A1, a 37 kDa member of the annexin super-family (13 proteins in mammals), and its N-terminal peptide N-acetyl_2-26_ [1,2], have been shown by us, and by other laboratories to possess powerful anti-inflammatory actions in a wide variety of animal models of acute [3–17] or chronic [18–20] inflammation.

The biologically active pool, in this context, is the extracellular protein. Anx-A1 is both induced and secreted from cells under the influence of GCs [21–24]. The release, as opposed to the induction, of cytosolic Anx-A1 is increased by GCs acting in a receptor-dependent, non-genomic manner. This GC-induced secretory event is preceded by Ser^27^ phosphorylation apparently as a result of PKC (Protein Kinase C; EC 2.7.11.13) activation [25–27]. Indeed, the Anx-A1 Ser^27^–Ala^27^ mutant is not secreted from cells and has a different intracellular distribution [28]. Once on the cell surface, Anx-A1 can act in an autocrine (or paracrine) fashion to inhibit cell activation probably by interaction with receptors of the FPR family, specifically FPR-L1 (ALXR; [29–31]).

The ‘cromoglycate-like’ anti-allergic drugs (cromolyns) are a group of compounds of which sodium cromoglycate and sodium nedocromil are the exemplars. The family also embraces lodoxamide, traxanol and amlexanox as well as some H_1_ antagonists such as ketotifen, azelastine, pemirolast and olopatidine, many of which appear to share a similar pharmacology (or exhibit cross-tachyphylaxis) with cromoglycate [32].

Contemporary reviewers are unanimous in attributing the anti-asthmatic activity of the cromoglycate-like drugs to their anti-inflammatory properties (see Refs. [33–35]), although the exact mechanism of action of this group of drugs has proved elusive. Early experiments [36–40] led to the concept that these drugs acted mainly on mast cells to suppress mediator release, but the balance of evidence now suggests that this is unlikely to be their only clinically significant action and that mast cells are not their sole target. Of particular relevance to this study is the observation that they can also suppress eicosanoid generation [41,42].

Here we report that the mechanism whereby the latter effect is accomplished in differentiated U937 cells depends upon the enhanced intracellular trafficking and release of Anx-A1, and that these drugs synergise strongly with agents which activate PKC, such as GCs, to bring about this effect. Our findings highlight a novel mechanism of action for the cromolyns as well as providing a compelling rationale for a combination anti-allergy therapy.

## 2 Materials and methods

### 2.1 U937 cell culture

U937 cells were obtained from the American Type Culture Collection and cultured in RPMI 1640 supplemented with 10% FCS, 1% l-glutamine, 1% non-essential amino acids and 0.1% gentamicin at 37 °C (Sigma–Aldrich, Poole, UK) in 5% CO_2_ atmosphere. Proliferating monocytic cells were removed from the flask when 70–80% confluence was achieved, transferred to 12-well plates at a density of 10^6^ cells/well and pre-incubated with 10 ng/ml phorbol 12-myristate 13-acetate (PMA) for 24 h to promote monocytic differentiation after that period they acquire sensitivity to GCs [43].

For assessment of drug effects, GCs were tested alone or in combination with the anti-allergic drugs for different times. Three different procedures were used to prepare samples for analysis. To analyse proteins of interest, U937 cells were normally lysed at the end of the treatment period and the total protein content was used for further analysis. To do this U937 cells in suspension following drug treatment, were decanted into 1.5 ml Eppendorf tubes and gently centrifuged (300 × *g*) for 5 min. The supernatant was removed and the resultant pellet resuspended in 500 μl of lysis buffer containing 1 mM EDTA (which removes Anx-A1 attached to cell membranes), 200 mM NaCl, 20 mM Tris–HCl (pH 8.0), 1 mM protease and 1 mM phosphatase inhibitors (equimolar mixture of Na_3_VO_4_, β-glycerophosphate, NaF) and 0.1% Triton-X.

In some experiments, where we studied the differential abundance of proteins in the cell cytosol and pellet, the cells were first ruptured by freeze-thawing in liquid nitrogen 3–4 times and the crude fractions separated by centrifugation at 13,000 × *g* for 5 min. The supernatant and pellet fractions were then prepared for analysis in lysis buffer as above prior to Western blotting.

To prepare U937 plasma membranes, cell organelles were separated by ultra-centrifugation. Cells ruptured by sonication were centrifuged at 300 × *g* for 5 min to remove coarse debris and intact cells and the supernatant was removed and resuspended in 1 ml lysis buffer. An initial centrifugation at 13,000 × *g* separated nuclei, mitochondria and other dense material. The supernatant from this step was then resuspended in 0.5 ml lysis buffer and centrifuged for 1 h at 100,000 × *g*. The resulting pellet was resuspended in lysis buffer containing 0.1% Triton-X.

The cellular protein content of different fractions was analysed to determine the total protein concentration using the BioRad Protein Assay Method (Bio-Rad Laboratories, Hemel Hempstead, UK) according to the manufacturer's instructions.

### 2.2 Transfection of U937 cells with GFP–Anx-A1 construct and fluorescence microscopy

U937 cells were stably transfected with a mouse Anx-A1 cDNA open reading frame-GFP as previously reported [28]. Cells transfected with EGFP ‘empty’ plasmid were used as controls. Stably transfected clones were kept in culture for no longer than 20 passages.

For transfection, the cells were plated in 6-well plates at a concentration of 10^6^/well in DMEM. Transient transfection was performed the following day, using a Dharmafect reagent 2 (Dharmacon-Perbio Science, Cramlington, UK), a liposoluble agent that fuses with the membrane, according to the manufacturer's protocol. These cells grow in suspension and after a period of 2 weeks in G418 selection, cells were sorted by FACS to enrich the GFP positive clones followed by a serial dilution in order to facilitate a clonal expansion.

To visualise export of Anx-A1–GFP cells were stimulated with GCs and/or cromoglycate-like drugs for 5–10 min, fixed in 4% paraformaldehyde (PFA), and stained with red fluorescent Alexa Fluor^®^ 594 wheat germ agglutin (WGA: Image-IT™ live plasma labelling kit, Molecular Probes, Leiden, The Netherlands) to visualise the plasma membrane of live Anx-A1–GFP transfected cells.

The slides were mounted in Mowiol (Sigma–Aldrich, Poole, Dorset, UK). Fluorescence micrographs were obtained with a Cool-Snap-Pro colour camera (Media Cybernetics, Finchampstead, Berkshire, UK) and image processing software (Image Pro Plus 4.5) linked to a Nikon Eclipse E800 microscope. The filter used to detect GFP-fluorescence was an excitation band-pass filter (450–490 nM), a dichroic mirror (510 nM), and an emission band filter (515–560 nM). Z-stack images at 0.5 μm separation were collected on an inverted epifluorescence TE2000 U Nikon microscope, and were subjected to nearest neighbour deconvolution using Openlab 5.5 software (Improvision, Coventry, UK).

### 2.3 Western blotting

The total cellular protein was determined and the supernatant analysed by conventional Western blotting techniques. Immunodetection was accomplished using different antibodies recognizing either the full-length Anx-A1 protein (polyclonal anti-Anx-A1 antibody; Invitrogen, Paisley, UK), Anx-A1 phosphorylated on Ser^27^ (polyclonal anti-Ser^27^–Anx-A1 antibody; Neosystem, Strasbourg, France) and α-tubulin (monoclonal anti-α-tubulin; Sigma–Aldrich, Poole, UK). A horseradish peroxidase-conjugated secondary antibody (Sigma–Aldrich, Poole, UK) detected bands related to the proteins of interest and these were revealed using ECL reagents and quantitated using the Image J densitometry program. All data were normalised to α-tubulin and expressed as percentage of control (differentiated cells stimulated with 10 ng/mL PMA for 24 h).

### 2.4 ELISA for Anx-A1

Anx-A1 protein levels in conditioned medium were determined by ELISA as reported by Goulding et al. [21]. Briefly, 96-well flat-bottomed ELISA plates (Greiner, Gloucestershire, UK) were coated with 1 μg anti-Anx-A1 mAb 1B in bicarbonate buffer (pH 9.6) and incubated overnight at 4 °C. After washing in the bicarbonate buffer, potentially uncoated sites were blocked with 100 μl of PBS containing 1% BSA for 1 h at room temperature. Sample aliquots (100 μl) or Anx-A1 standard solutions (prepared in 0.1% Tween-20 in PBS; concentration ranging between 10 and 0.001 μg/ml) were added for 1 h at 37 °C. After extensive washing in PBS/Tween-20, 100 μl of a polyclonal rabbit anti-human Anx-A1 serum (Zymed cat no 71–3400, Invitrogen, Paisley, UK; diluted 1:1000 in PBS/Tween-20) was added (1 h at 37 °C) before incubation with donkey anti-rabbit IgG conjugated to alkaline phosphatase (1:1000; Sigma–Aldrich, Poole, UK). The colour was developed by addition of 100 μl p-nitrophenyl phosphate (pNPP) (Sigma–Aldrich, Poole, UK; 1 mg/ml in bicarbonate buffer, pH 9.6). Absorbance was read at 405 nm (with a 620-nm reference filter) in a microplate reader (Titertek™, Vienna, Austria). Anx-A1 levels in the study samples were read against the standard curve and expressed as ng/ml.

### 2.5 Electron microscopy

After drug treatment as described above, U937 cells were fixed with a mixture of freshly prepared 3% (w/v) paraformaldehyde and 0.5% (v/v) glutaraldehyde in PBS (pH 7.2) for 4 h at 4 °C, washed briefly in PBS, and transferred to a solution of 2.3 M sucrose (in PBS) at 4 °C overnight. The cyroprotected cells were slam-frozen (Reichert MM80E; Leica, Milton Keynes, UK), freeze-substituted at −80 °C in methanol for 48 h, and embedded at −20 °C in LRGold acrylic resin (Agar Scientific, Stansted, UK) in a Reichert freeze-substitution system. Ultrathin sections (50–80 nm) were prepared using a Reichert Ultracut-S ultratome and incubated at room temperature for 2 h with a well-characterised in-house polyclonal sheep anti-Anx-A1 antibody (dilution 1:200) followed by a second antibody labelled with immunogold (British Biocell, Cardiff, UK). The serum and antiserum were diluted in 0.1 M phosphate buffer containing 0.1% egg albumin. After immunolabelling, sections were lightly counterstained with uranyl acetate and lead citrate (British Biocell, Cardiff, UK) and examined with a JEOL 1010 transmission electron microscope (JEOL, Peabody, MA).

The number of cytoplasmic and membrane 15 nm gold particles were counted in 30 cells and calculated as particles/μm^2^ by dividing the total number of gold particles counted by the cell area. For measurement of cell area micrographs of each cell were taken at a magnification of 4000×. The cell areas were analysed from scanned micrographs using Axiovision software, version 3.4 (Zeiss, Hemel Hempstead, UK). In all cases the analyst was blind to the sample code.

### 2.6 Measurement of TxB_2_ activity

Supernatants (2 ml) from individual samples were concentrated (10×) using Centricon centrifugal filter devices (Millipore, Watford, UK) with Ultracel YM-10 membrane centrifuged at 5000 × *g* for 1 h. An enzyme immunoassay was established to detect and quantify TxB_2−_ released in the supernatant (Biotrak Assay, Amersham, UK). The method was conducted following the manufacturer protocols. A standard curve ranging from 0.5 to 64 pg/well was prepared using the reagent provided and the optical density was then read at 450 nm in a microplate reader, within 30 min.

### 2.7 PKC activity assay

The assay was performed using the PKC Kinase Activity Assay Kit (Stressgen, Cambridge Bioscience, Cambridge, UK) as described in the manufacturer's protocol: each sample was loaded on to a pre-coated plate with a substrate peptide for PKC and the reaction initiated by adding ATP. The phosphospecific substrate antibody (rabbit polyclonal) was added and detected by an HRP-conjugated anti-rabbit IgG and the colour developed with a TMB substrate in proportion to PKC phosphotransferase activity. The reaction was stopped with 100 μl of 1 M H_2_SO_4_ and the colour was measured on a microplate reader at 450 nm. The kinase activity in the cell lysate was calculated as a ratio between the average of absorbance in each sample (subtracted by the absorbance in the blank) and the amount of protein loaded per assay. A recombinant active protein kinase C was used as a positive control.

### 2.8 Phosphatase activity assay

To detect protein phosphatase (phosphoprotein phosphatase; EC 3.1.3.16) activity in the samples, we used the colorimetric Sensolyte pNPP Protein Phosphatase kit (ANASPEC, San Jose, CA, USA). Membrane or recombinant PP2A samples were prepared according to the protocols suggested by the manufacturer. The colorimetric substrate p-nitrophenyl phosphate was used to assess the activity of generic phosphatase activity in our samples, yielding a yellow colour that can be quantified at 405 nm from which the substrate hydrolysis calculated from the molar extinction coefficient supplied. For a kinetic reading, the absorbance was measured every 5 min for 30 min. Samples containing drug alone without enzyme were monitored to check that they had no influence on the colour reaction.

### 2.9 Drugs and materials

The following chemicals (EDTA, glutaraldehyde, β-glycerophosphate, H_2_SO_4_, methanol, NaCl, NaF, Na_3_VO_4_, paraformaldehyde, PMA, sucrose, Tris–HCl and 0.1% Triton-X) and drugs (betamethasone, dexamethasone, hydrocortisone, 5-methylprednisolone and prednisolone, PI3 kinase inhibitor (LY 294002), MAP kinase inhibitor (PD98059), mifepristone (RU 486), okadaic acid and di-sodium cromoglycate) were purchased from Sigma–Aldrich, Poole, Dorset, UK. Highly purified (>90%) bovine PP2A 1800.0 U/mg was obtained from Calbiochem (Merck Chemicals, Nottingham, UK). Sodium nedocromil was a generous gift from Sanofi-Aventis.

All drugs were diluted in incubation medium immediately before use to a final concentration that did not exceed 0.04% (w/v).

### 2.10 Data analysis

For electron microscopy, all values for immunogold particles counted represent the mean ± S.E.M.: *n* = 30 cells per group. Preliminary analysis confirmed that the data were normally distributed. Subsequent analysis was undertaken by one-way analysis of variance (ANOVA) with post hoc analysis performed using Scheffe's test.

Elsewhere, all data are presented as mean ± S.E.M. and were tested for normality prior to analysis. Statistical differences between groups were established using one-way ANOVA followed by Bonferroni post hoc test.

In all cases differences were considered significant if *P* < 0.05.

## 3 Results

### 3.1 GCs alone stimulate Anx-A1 phosphorylation through a PKCα/β-dependent mechanism

GCs increase both the synthesis (genomic) as well as the release (non-genomic) of Anx-A1 from cells. As most previous studies have focussed upon the former we determined, in a series of pilot studies, the time course of GC action on these separate processes in U937 cells (data not shown). Treatment with 1 μM dexamethasone strongly stimulated the production of the Ser^27^ phosphorylated species within 5 min and this was sustained for up to 30 min. There were no changes in the total mass of Anx-A1 protein in cells prior to the 60 min time point after which steadily increasing amounts accumulated during the following 24 h. We therefore chose 5 min as the optimal time point for most of our subsequent assays as strong non-genomic effects of the GC could be easily observed without detectable genomic actions occurring. We tested dexamethasone, hydrocortisone, prednisolone, methylprednisolone and betamethasone in this system finding that all stimulated Anx-A1 Ser^27^ phosphorylation in a qualitatively comparable fashion, however prednisolone, methylprednisolone and betamethasone were not examined further in our assay systems.

**Fig. 1:**
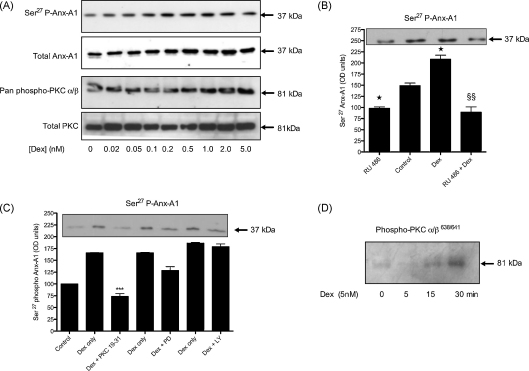
Dexamethasone increases the phosphorylation of Anx-A1 and PKC in U937 cells within 5 min in a concentration-dependent fashion. (Panel A) In U937 cells, 5 min treatment with dexamethasone in concentrations of 0.02–5.0 nM increases phosphorylation of Anx-A1 on Ser^27^ (upper panel) by ∼4-fold and, in parallel, PKCα/β phosphorylation (lower panel) by ∼3-fold compared to untreated controls. Whole U937 cell lysates were prepared as detailed. Anx-A1 phosphorylation was detected using a specific anti-Ser^27^ phospho Anx-A1 antibody as described. Total Anx-A1 (sometimes shown as a 33/37 kDa doublet) and PKC is shown for reference purposes. (Panel B) The dexamethasone (2 nM) effect on Anx-A1 phosphorylation is dependent on GC receptor occupation since the co-administration of 1 μM RU 486, blocks this effect. Note that the amount of phospho Anx-A1 is significantly less in samples treated with RU 486 alone than in vehicle treated samples, suggesting that some residual GCs may be present in the culture media. *P < 0.05 relative to control values; ^§§^*P* < 0.01 relative to dexamethasone alone. (Panel C) The action of common kinase inhibitors on Anx-A1 phosphorylation. Of these, only PKC 19–31 (5 μM), an inhibitor of PKC, was able to reduce phosphorylation of Anx-A1 when this was stimulated by 2 nM dexamethasone (D). An inhibitor of PI3 kinase inhibitor LY294002 (LY: 10 nM) or a MAP kinase inhibitor PD98059 (PD: 5 μM) were inactive. Insert: a representative Western blot showing effect of the different treatments on Ser^27^ phospho Anx-A1. ***P < 0.001 relative to dexamethasone alone. (Panel D) Analysis of the PKC isoform activated by GCs. Blots were probed with anti-sera specific for each isoform. No signal was seen with antisera recognising PKC θ (not shown). A faint signal was detected for PKC δ Thr^55^ (78 kDa) but not for its activated form, Ser^643^ (not shown). In contrast, activated PKCα/β Thr^638/641^ (81 kDa), showed a strong signal in response to dexamethasone treatment at 15–30 min. All experiments were performed at least three times and the blots are representatives from one of these experiments. Densitometry was performed as described in the methods and the optical density units normalised by comparison to α-tubulin. Data are expressed as mean ± S.E.M. Statistical differences between groups were established using one-way analysis of variance (ANOVA) followed by Bonferroni post hoc test.

We then established the concentration dependency of GC-induced Anx-A1 phosphorylation in U937 cell lysates at 5 min. Fig. 1A (upper panel) shows that increasing concentrations of dexamethasone (0.02–5.0 nM) produced a corresponding augmentation (∼4-fold, by densitometry) of Anx-A1 Ser^27^ phosphorylation. We examined the concentration–response curves of dexamethasone, prednisolone and hydrocortisone in detail and determined the relative EC_50_ concentrations to be 0.2, 0.4 and 1 nM respectively. In this paper we report data obtained mainly using dexamethasone but we observed that hydrocortisone exhibited qualitatively identical behaviour in terms of its interaction with the cromolyns.

Previous studies have implicated the enzyme PKC in this GC-induced phosphorylation of Anx-A1. We tested this in the present system using a pan-specific anti-phospho PKC antibody, finding that the abundance of phosphorylated PKC was increased in a similar concentration-dependent fashion (>3-fold; Fig. 1A lower panel) by these drugs to that of Ser^27^ phospho Anx-A1.

To confirm that the GC effect was dependent upon GR occupation we tested the effect of the GR antagonist RU 486 on the ability of dexamethasone to stimulate phosphorylation of Anx-A1. Fig. 1B shows that 2 nM dexamethasone was unable to stimulate Anx-A1 Ser^27^ production in the presence of 1 μM of this drug (*P* < 0.01). It is noteworthy that the background Anx-A1 phosphorylation sometimes observed in untreated cells was itself reduced by RU 486 (*P* < 0.05) perhaps implying that residual GCs in the medium may drive a low level phosphorylation of the protein.

To determine if PKC was the sole kinase responsible for Anx-A1 phosphorylation in this system, we examined the effects of a panel of inhibitors. Fig. 1C shows the effect of the PI3 kinase inhibitor LY294002 (10 nM), a MAP kinase inhibitor PD98059 (5 μM) and an inhibitor of PKC, PKC 19–31 (5 μM) on the phosphorylation response. Only PKC 19–31 was able significantly (*P* < 0.001) to reverse the dexamethasone-induced increase in Anx-A1 phosphorylation. The MW of the phosphorylated PKC enzyme detected by Western blotting using the pan-specific PKC antibody was in the range 76–82 kDa suggesting the relevant isoform was either PKC δ (78 kDa), PKC θ (76 kDa) or PKCα/β (80–82 kDa). When we probed our blots with a panel of isoform specific anti-phospho PKC antisera, no PKC δ (Ser^643^) or PKC θ Thr^538^ was detectable. Some PKC δ Thr^505^ were detected but with a lower MW (78 kDa) than the GC stimulated species seen in our blots. However, the specific anti-phospho-PKCα/β Thr^638/641^ antibody showed good reactivity with a band that increased with GC treatment over a period of 5–30 min treatment (Fig. 1D). We therefore concluded that the main kinase responsible for Anx-A1 phosphorylation in this system was almost certainly PKCα/β.

### 3.2 Cromoglycate-like drugs synergise with GCs to stimulate Anx-A1 phosphorylation and release

**Fig. 2:**
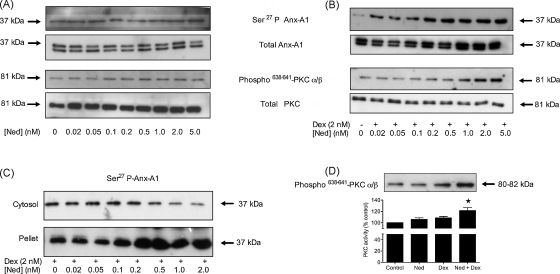
Nedocromil potentiates the effect of dexamethasone on Anx-A1 and PKCα/β phosphorylation and protein translocation to the plasma membrane fraction. (Panel A) Nedocromil itself, over a range of concentrations, has a negligible effect on the concentration of Ser^27^ phospho Anx-A1 in U937 cell lysates or PKCα/β phosphorylation when compared to untreated samples. Total Anx-A1 (sometimes shown as a 33/37 kDa doublet) and PKC is shown for reference purposes. (Panel B) In the presence of 2 nM dexamethasone, escalating concentrations (0.02–5.0 nM) of nedocromil potentiate (by a further 3–4-fold) the phosphorylation of both Anx-A1 and PKCα/β. Total Anx-A1 (sometimes shown as a 33/37 kDa doublet) and PKC is shown for reference purposes. (Panel C) In the presence of a fixed concentration of dexamethasone (2 nM), nedocromil (0.02–2.0 nM) potentiates the translocation of Ser^27^ phospho Anx-A1 from the cytosol into the 13,000 × *g* particulate fraction of U937 cells. (Panel D) The membrane accumulation of activated PKCα/β is promoted (>2-fold) by the combination of nedocromil (0.5 nM) and dexamethasone (2 nM; see blot insert) and this is reflected in an increase in enzyme activity as measured in the 100,000 × *g* membrane fraction. *P < 0.05 relative to nedocromil of dexamethasone alone. All experiments were done at least three times and the blots are representatives from one of these experiments. Densitometry was performed as described in the methods and the optical density units normalised by comparison to α-tubulin. Data are expressed as mean ± S.E.M. Statistical differences between groups were established using one-way analysis of variance (ANOVA) followed by Bonferroni post hoc test.

We next assessed the effect of sodium cromoglycate and nedocromil on Anx-A1 and PKCα/β phosphorylation in our U937 cell system. Fig. 2A shows that the administration of nedocromil (0.02–5.0 nM) alone had a negligible or only a weak action on the phosphorylation of Anx-A1 (<1.5-fold) or PKCα/β (1–2-fold) when compared with dexamethasone (see Fig. 1A and B). Cromoglycate alone (0.02–5.0 nM) was similarly only very weakly active in this system (data not shown). Surprisingly however, in the presence of a fixed concentration (2 nM) of a GC, these drugs exhibited a further strong concentration-dependent increase in both Anx-A1 and PKCα/β phosphorylation (>3–4-fold; Fig. 2B) that exceeded the maximum stimulation caused by the GCs alone. In this paper we mainly report data obtained using nedocromil but we observed that cromoglycate exhibited qualitatively the same effect in all our assay systems.

### 3.3 Translocation of phosphorylated species

PKCα/β is enriched at the plasma membrane when activated and Anx-A1 is likewise recruited to the membrane upon phosphorylation at Ser^27^. Fig. 2C shows that in the presence of a fixed concentration of dexamethasone, escalating concentrations of nedocromil cause the translocation of Ser^27^ phospho Anx-A1 from the (13,000 × *g*) supernatant to the particulate fraction of the U937 cells. We refined this analysis by using differential centrifugation to prepare some 100,000 × *g* membranes from cells treated with drug combinations. Fig. 2D shows that, relative to either drug given alone, the combination of nedocromil and dexamethasone increased (2-fold) the amount of activated phospho PKCα/β in the membrane fraction as determined by Western blotting and also increased (*P* < 0.05) the catalytic activity of the enzyme in this compartment.

### 3.4 Cromoglycate-like drugs potentiate the action GCs on eicosanoid release

The ability of GCs to arrest eicosanoid synthesis depends on the release of Anx-A1 from target cells in many systems. As the cromoglycate-like drugs are also reported to inhibit eicosanoid formation, we next determined whether these drugs alone, or in combination with GCs, enhanced Anx-A1 release thereby producing a concomitant decrease in the generation of TxB_2_, a prominent eicosanoid spontaneously released from U937 cells.

**Fig. 3:**
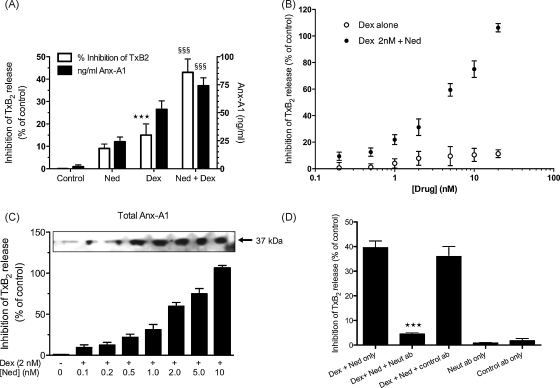
Nedocromil potentiates dexamethasone inhibition of TxB_2_ release from U937 cells. (Panel A) Measurement of TxB_2_ generation (left hand *Y*-axis) and Anx-A1 (right hand *Y*-axis) by ELISA assay in the medium of U937 cells after treatment with nedocromil (5 nM), dexamethasone (2 nM), or a combination of both. The varying inhibition of TxB_2_ release corresponds with the amount of Anx-A1 externalised. ***P < 0.001 relative to control values; ^§§§^*P* < 0.001 relative to dexamethasone alone values. (Panel B) Dexamethasone itself (0.2–20.0 nM) inhibits TxB_2_ release at 5 min (as assessed by ELISA assay) but does not produce a maximal effect. However, when nedocromil (0.05–20 nM) is added to a maximally effective (2 nM) concentration of dexamethasone, a concentration-dependent potentiation of the inhibitory effect occurs with near maximal inhibition achieved at 20 nM nedocromil. (Panel C) The inhibition of TxB_2_ release (as assessed by ELISA assay) by escalating concentrations of nedocromil (0.2–10 nM) in the presence of a fixed concentration (2 nM) of dexamethasone is paralleled by increasing amounts of Anx-A1 externalised from U937 cells. Insert: representative Western blot in which the total Anx-A1 in the medium was assessed. (Panel D) Reversal of the TxB_2_ inhibitory action of a combination of 2 nM dexamethasone with 0.5 nM nedocromil by a mouse anti-Anx-A1 neutralising monoclonal antibody (mab 1A) but not by an irrelevant isotype matched monoclonal antibody. ***P < 0.001 relative to dexamethasone + nedocromil values. All experiments were done at least three times and the blot is representatives from one of these experiments. Densitometry was performed as described in the methods and the optical density units normalised by comparison to α-tubulin. Data are expressed as mean ± S.E.M. Statistical differences between groups were established using one-way analysis of variance (ANOVA) followed by Bonferroni post hoc test.

Fig. 3A shows that whilst nedocromil (0.5 nM) alone had only a small (<10%; NS) effect on TxB_2_ release it greatly potentiated (*P* < 0.001) the inhibitory effect of 2 nM dexamethasone (*P* < 0.01) alone. This correlated with a potentiation of Anx-A1 release into the medium as assessed by the ELISA assay (*P* < 0.001).

Fig. 3B and C shows the concentration dependency of this effect. In Fig. 3B, the maximum inhibition achieved by dexamethasone at 5 min (<20%: 0.2–20 nM) alone was evident at a concentration of 1 nM. When nedocromil (0.2–20 nM) was added to 2 nM dexamethasone however, a concentration-dependent increase in the inhibitory activity was observed which maximally inhibited TxB_2_ release at concentrations of 20 nM nedocromil.

Fig. 3C shows that this is also true of Anx-A1 release. In the presence of 2 nM dexamethasone, nedocromil (0.2–10.0 nM) caused a concentration-dependent inhibition of TxB_2_ generation that was correlated exactly with the increasing amounts (7–8-fold) of Anx-A1 released onto the outside of the cell (see blot insert).

To confirm that the drug-induced inhibition of TxB_2_ was caused entirely by increased Anx-A1 in this system we tested the effect of a neutralising anti-Anx-A1 monoclonal (mab 1A) and isotype-matched irrelevant monoclonal antibody. Fig. 3D shows that addition of the mabs alone had no effect but that the neutralising, but not the irrelevant monoclonal antibodies completely reversed (*P* < 0.001) the inhibitory effect of the dexamethasone–nedocromil combination on TxB_2_ release.

### 3.5 GCs and cromoglycate-like drugs promote intracellular trafficking and membrane localisation of Anx-A1 in U937 cells

To investigate the effect of the drugs on the intracellular movement of Anx-A1 at a fine level of detail, we utilised U937 cells that had been transfected with an Anx-A1–GFP construct and analysed the effects of drug treatment by confocal microscopy.

**Fig. 4:**
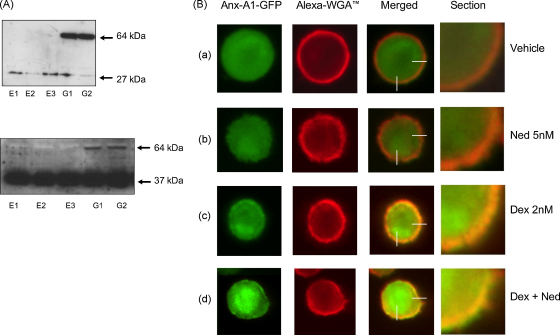
The effect of nedocromil and dexamethasone on the disposition of Anx-A1 in U937 cells transfected with an Anx-A1–GFP construct. (Panel A, upper figure) The Western blot, probed with an anti-GFP antibody, shows that only GFP protein (27 kDa) was seen in U937 cells transfected with the mock plasmids E-1, E-2 and E-3 whereas in U937 cells transfected with Anx-A1–GFP constructs G-1 and G-2 a 64 kDa band corresponding to the GFP–Anx-A1 conjugate was observed. Clones G-1, G-2, E-1 and E-3 were used for this study. (Lower figure) This blot shows the complementary experiment to that in panel A, where the samples were probed with the anti-Anx-A1 antibody. (Panel B) In these experiments, the plasma membrane of U937 cells transfected with the Anx-A1–GFP construct, was stained red with Alexa Fluor^©^ 594 WGA dye and the cells subjected to various drug treatments protocols. The four columns represent the information from the two colour channels, the merged channel and a magnified (90×) section of the membrane. (Row a) The situation when the transfected U937 cells are treated with vehicle alone. Anx-A1 is mainly contained within the cytoplasmic compartment and the plasma membrane stains red. (Row b) There appears to be little evidence of co-localisation after treatment with 5 nM nedocromil alone. (Row c) Treatment with dexamethasone alone 2 nM clearly promotes co-localisation of Anx-A1 and membrane phospholipids. (Row d) Treatment with dexamethasone and nedocromil in combination leads to a striking co-localisation of Anx-A1 with the membrane and evidence of release of GFP-tagged protein into the external medium. Magnification 90×. This figure is representative of three independent experiments.

The Western blots in Fig. 4A shows that only GFP protein (27 kDa) was seen in U937 cells transfected with the mock plasmids E1, E2 and E3 whereas in U937 cells transfected with Anx-A1–GFP constructs G1 and G2 a 64 kDa band corresponding to the GFP–Anx-A1 conjugate was observed. Clones G1, G2, E1 and E3 were selected for this study (upper panel probed with anti-GFP and lower with anti-Anx-A1 antibodies).

Fig. 4B shows an experiment where we used Anx-A1–GFP transfected U937 cells in which the plasma membrane has been stained with the red fluorescent Alexa Fluor^®^ 594 wheat germ agglutinin (WGA) labelling reagent that specifically stains membrane phospholipids. The Anx-A1–GFP in vehicle treated cells is localised in the cytoplasm (row a). Not much change is seen with nedocromil (5 nM) treatment alone (row b), however, treatment with dexamethasone (2 nM) alone induces some degree of Anx-A1 redistribution, as shown by a partial co-localisation of Anx-A1 with the membrane (row c, merged image). When dexamethasone is combined with 5 nM nedocromil, however a striking degree of co-localisation of Anx-A1 with the membrane is observed (row d) and evidence of membrane ‘bubbling’ (possible related to secretory activity) may be inferred from the presence of GFP–Anx-A1 outside the cell membrane.

Although we observed that the non-genomic actions of these drugs were evident at 5 min, we were curious about events that might take place over a shorter time-span. To investigate this we used a slightly different experimental design in which time-lapse video-microscopy was used. GFP-tagged Anx-A1 transfected U937 cells were filmed for 3 min under various experimental conditions, and the frames examined for changes that might reflect the release of Anx-A1 in response to drugs.

**Fig. 5:**
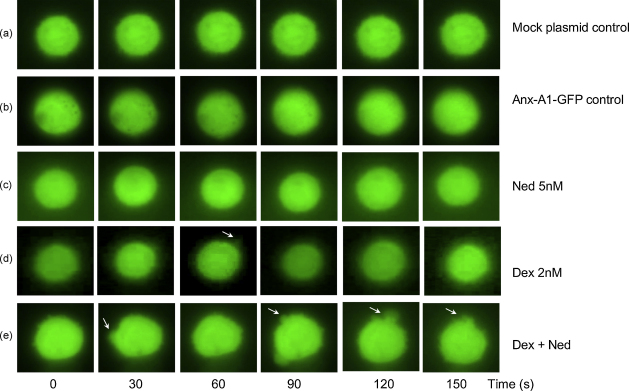
Time-lapse photography of U937 cells transfected with the Anx-A1–GFP construct after treatment with nedocromil and dexamethasone. Individual frames (6 frames, 30 s intervals) were taken from time-lapse video-microscopy of 3 min duration during which transfected U937 cells were exposed to different experimental treatments. (Row a) Cells transfected with mock plasmid and vehicle only. (Row b) Cells transfected with Anx-A1–GFP plasmid and treated with vehicle alone. (Row c) Cells transfected with Anx-A1–GFP plasmid and treated with nedocromil (5 nM) alone. (Row d) Cells transfected with Anx-A1–GFP plasmid and treated with dexamethasone (2 nM) alone. Note evidence of some secretory activity in one frame at 60 s (arrowed). (Row e) Cells transfected with Anx-A1–GFP plasmid and treated with a combination of dexamethasone (2 nM) and nedocromil (5 nM). Notice that the extrusion of GFP–Anx-A1 (arrowed) is seen within 30 s of drug administration and becomes extremely pronounced, at multiple sites on the membrane, by 2 min. Magnification 90×. This figure is representative of three independent experiments.

Fig. 5 shows a panel of individual 6 frames taken at 30 s intervals throughout these experiments during which drug (or vehicle) treatments were administered. Treatment of cells with vehicle or nedocromil (5 nM) alone (rows b and c) had little or no discernable effect. Treatment with dexamethasone (2 nM) alone produced some evidence of secretory activity at 60 s congruent with our other data. However, when a combination of dexamethasone and 5 nM nedocromil was administered, the GFP-tagged protein was released from focal sites on the membrane within 30 s and release from multiple sites was very pronounced within 3 min.

### 3.6 Electron microscopy studies

**Fig. 6:**
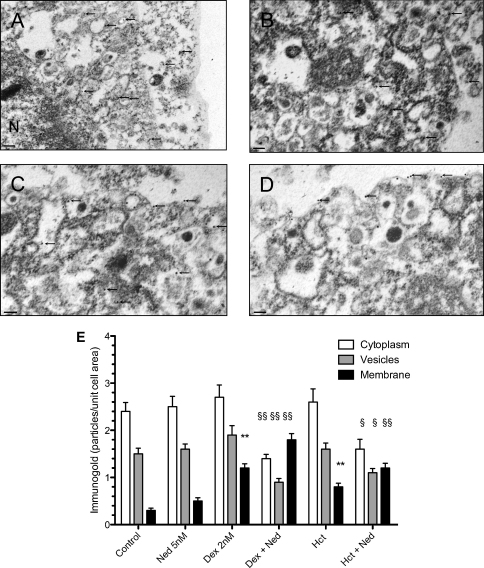
Electron microscopy of U937 cells after treatment with dexamethasone and/or nedocromil. (Panel A) Vehicle treatment. U937 cells treated with vehicle alone for 5 min were fixed and processed as described in materials and methods. In this image (note a slightly lower power than the others to provide an overview of distribution) immunogold labelled Anx-A1 (arrowed) was mainly to be found in the peri-nuclear region or in the cytoplasm apparently associated with vesicles (arrowed). Little was seen on the plasma membrane. N; nucleus. Scale bar 0.5 μm. (Panel B) After exposure to 5 nM nedocromil alone, little change was seen in the distribution of immunogold labelled Anx-A1 (arrowed) in the cells. The protein was once again found chiefly in the cytoplasm, mainly in conjunction with vesicles with only minimal membrane labelling. Scale bar 0.1 μm. (Panel C) Dexamethasone (2 nM) treatment provoked some secretory activity with immunogold labelled Anx-A1 being identified on the plasma membrane as well within cytosolic vesicles. Scale bar 0.1 μm. (Panel D) After simultaneous treatment with both 5 nM nedocromil and 2 nM dexamethasone, substantial amounts of immunogold labelled Anx-A1 had translocated to the cell membrane. Scale bar 0.1 μm. (Panel E) Quantitation of immunogold labelled Anx-A1 by electron microscopy showing the effect of combinations of 5 nM nedocromil and dexamethasone or hydrocortisone (2 nM) on the intracellular distribution of Anx-A1. Note the profound effect of the combination of the two different types of drugs. Data is presented as mean ± S.E.M. and are representative of three replicate experiments (*n* = 30 cells per group) all of which were processed separately. Subsequent analysis was undertaken by one-way ANOVA with post hoc analysis performed using Scheffe's test. **P < 0.01 membrane Anx-A1 increased relative to control values. ^§^*P* < 0.05, ^§§^*P* < 0.02 Anx-A1 changed relative to control values.

The experiments with the transfected U937 cells could be compromised by the fact that Anx-A1 was conjugated to GFP and therefore that its intracellular trafficking might be atypical. To address this we used conventional electron microscopic and immuno-chemistry techniques to follow the fate of Anx-A1 in response to various drug treatments. Fig. 6A–D shows the results of this investigation together with a graphical analysis of the quantitation of immunogold particles in Fig. 6E. In untreated U937 cells immunogold labelled Anx-A1 was found predominantly in the peri-nuclear region and the cytosol, often in combination with vesicular structures (panel 6A) with little discernable at the membrane. Nedocromil (5 nM) had little effect on this pattern of distribution (panel 6B) but dexamethasone (2 nM; panel 6C) or hydrocortisone (2 nM) promotes association of the protein with the membrane (*P* < 0.01). In the presence of both GCs and nedocromil however (panel 6D), there was a significant (*P* < 0.01) increase of membrane-associated Anx-A1 and a corresponding decrease in cytosolic and vesicular associated material.

### 3.7 Cromoglycate-like drugs inhibit membrane phosphatase activity

All the data generated so far pointed to a mechanism whereby Anx-A1, translocated to the membrane after phosphorylation on Ser^27^ under the influence of activated PKCα/β, was released from the cell to exert an extracellular inhibitory effect on eicosanoid synthesis and that this process was somehow potentiated by the cromoglycate-like drugs.

**Fig. 7:**
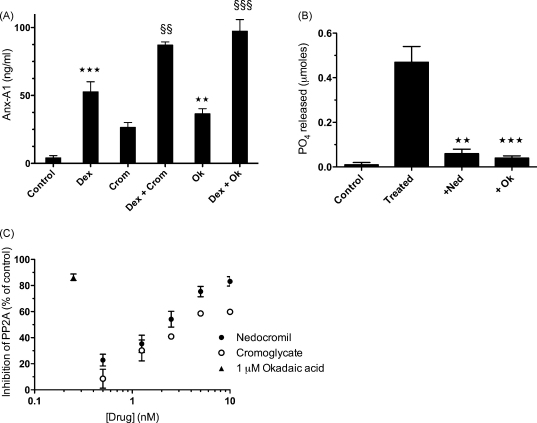
The cromoglycate drugs inhibit PP2A phosphatase activity. (Panel A) Okadaic acid (1 μM) as well as cromoglycate (2 nM) potentiate the action of 2 nM dexamethasone on Anx-A1 release from U937 cells as assessed by ELISA assay of the cell medium. ***P < 0.001, **P < 0.01 relative to control values; ^§§§^*P* < 0.001, ^§§^*P* < 0.01 relative to dexamethasone only. (Panel B) Nedocromil (2 nM) and okadaic acid (1 μM) inhibit the enhanced phosphatase activity of 100,000 × *g* observed in membranes prepared from dexamethasone-treated U937 cells. The drugs were pre-incubated with the membranes for 5 min before being added to the phosphatase assay plate and the hydrolysis assessed after 10 min. ***P < 0.001, **P < 0.01 relative to treated values. (Panel C) Nedocromil and cromoglycate inhibit the activity of a highly purified bovine PP2A enzyme in a concentration-dependent manner. The drugs were pre-incubated with the enzyme for 5 min before being added to the phosphatase assay plate and the hydrolysis assessed after 10 min. The IC_50_ values are nedocromil 0.65 nM and cormoglycate 1.7 nM respectively. 1 μM okadaic acid, included as a positive control in this assay, inhibited the reaction by >80%. Wells in which the drugs were incubated with the assay reagents alone indicated that they did not interfere with the development of the colour reaction. All experiments were performed at least three times. Data are expressed as mean ± S.E.M. Statistical differences between groups were established using one-way analysis of variance (ANOVA) followed by Bonferroni post hoc test.

The catalytic activity of PKCα/β is limited by membrane phosphatases, in particular PP2A, and we hypothesised that the cromoglycate-like drugs inhibited the activity of these enzymes thus prolonging the catalytic action of PKCα/β and indirectly potentiating the generation and release of Ser^27^ phospho Anx-A1. Since okadaic acid is a well-characterised inhibitor of PP2A (and PP1 in higher concentrations), we investigated its action in the U937 system. Fig. 7A shows that this inhibitor (1 μM) does indeed (*P* < 0.001) share the ability of cromoglycate to potentiate Anx-A1 release in this system.

We next measured the net phosphatase activity in U937 100,000 × *g* membranes. In the experiment depicted in Fig. 7B, we prepared 100,000 × *g* membranes from U937 dexamethasone-treated cells (as described above) and then pre-incubated them for 5 min with either 5 nM nedocromil or 1 μM okadaic acid before assessing their phosphatase activity at 10 min (the time point that gave maximal readings in pilot studies; data not shown). Strikingly, phosphatase activity was almost completely inhibited in the presence of either nedocromil or okadaic acid.

Finally, we tested the effect of cromoglycate and nedocromil on a highly purified PP2A preparation from bovine kidney. Fig. 7C shows that both nedocromil and cromoglycate inhibited the catalytic activity of this enzyme in a concentration-dependent manner after pre-incubation with the phosphatase for 5 min with IC_50_s of approximately 0.65 and ∼1.7 nM respectively. As expected, okadaic acid was also strongly inhibitory (IC_50_ ≪ 1 μM).

## 4 Discussion

The Anx-A1 system in undifferentiated myelomonocytic U937 cells does not respond to glucocorticoids and it is necessary to pre-treat the cells with low concentrations of PMA to render them responsive [43]. Using this model system, we have studied the rapid non-genomic effects of GCs on Anx-A1 intracellular trafficking and export and investigated the eicosanoid inhibitory mechanism of action of the cromoglycate-like drugs.

Concerning the former, we have confirmed and extended earlier reports (e.g. Refs. [25,27]) that GCs exhibit two distinct actions on the synthesis and intracellular distribution of Anx-A1 with a rapid non-genomic, receptor-dependent, action seen within 5 min of treatment followed by a ‘slow’ genomic action of GCs with a much longer latency, as judged by the increasing intracellular mass of Anx-A1 seen at time points longer than 1 h. We have also confirmed a mandatory role for Ser^27^ phosphorylation of Anx-A1 prior to membrane translocation and export and established that the kinase most likely to drive this process is the PKCα/β isoform of PKC.

GC effects in U937 cells were evident within minutes demonstrating how rapidly these agents can act through the Anx-A1 system. Whilst the ‘classical’ genomic actions of GCs have been considered to take hours to develop, there is increasing evidence in a number of systems (e.g. Refs. [44–47]) that these rapid non-genomic effects, including many which require GR ligation (e.g. Refs. [48,49]) are common, and may be crucial to the homeostatic or therapeutic action of these drugs. Interestingly, we observed some ‘background’ Anx-A1 phosphorylation in our cultured U937 cells in the absence of GC stimulation. At least part of this effect may have caused by endogenous GCs in the culture media since it was reduced in the presence of RU 486—and may have been reduced even further if charcoal-stripped serum had been used. However, stimuli other than GCs can also cause Anx-A1 phosphorylation [28], which often appears in response to cellular ‘stress’. *In vivo*, where there is a constant GC ‘tone’, one would anticipate some action of the cromolyns without further stimulation.

Our novel finding that the cromoglycate-like drugs greatly potentiated the effect of GCs on Anx-A1 release and eicosanoid inhibition throws interesting light upon the way in which this anti-inflammatory protein is externalised as well as providing the basis for a novel mechanistic explanation for the eicosanoid inhibitory activity of these anti-allergic drugs. According to this new paradigm for cromolyn action, we propose that these drugs act by potentiating the release of the anti-inflammatory protein Anx-A1 from cells. They accomplish this by promoting phosphorylation of Anx-A1 but only when this has been triggered by GCs or some other stimulus such as cell activation. This is amply demonstrated by the imaging techniques and the EM studies which are in excellent agreement with the Western blotting and ELISA data concerning the fate of Anx-A1 following treatment with these drugs.

Anx-A1 is present in several subcellular compartments of cells including the nucleus, the cytoplasm, the plasma and other membranes as well as attached to the cytoskeleton [22,50,51]. It has been repeatedly observed that ‘activation’ of macrophages and other cells can lead to an accumulation of Anx-A1 at the plasma membrane where it can (for example) block EGF [25] and LPS induced ERK activation [52] and undoubtedly fulfil other intracellular roles (e.g. Refs. [50,53,54]).

The actual mechanism of Anx-A1 secretion itself has not been addressed in this paper and remains unclear. The protein does not contain a canonical signal sequence and it is generally accepted that secretion occurs through a non-classical pathway. There is some evidence that the ABC transporter system could be involved [55–57] but other potential mechanisms could include a ‘diffusion controlled’ process facilitated by binding of the exported species to extracellular proteoglycans as suggested for fibroblast growth factor 2 [58]. A recent paper suggests that ceramide platforms in cells may preferentially bind Anx-A1 and could be important for externalisation [59].

Whichever mechanism operates, phosphorylation of the protein at Ser^27^ is clearly a crucial prelude to membrane recruitment and subsequent export and so it is probable that any stimulus that activates PKCα/β will lead to the marginalisation and release of Anx-A1 from cells in which this signalling system is supported. However, it is also evident that not all marginalised Anx-A1 is immediately exported suggesting a further control over this process acting at the level of the membrane itself.

Several authors have reported that GC receptor ligation activates PKC thus promoting its translocation to the plasma membrane (see Refs. [60–63]). Previous work from our group suggests that in some systems, phosphatidylinositol 3-kinase may play a key role [27] in activating PKC. PKC activity is ultimately terminated by dephosphorylation at the membrane probably by the Ser/Thr PP2A phosphatase [64] and ubiquitination. In many cell types, including the macrophage, PKCα (and some other isoforms) activate (and may associate with) PP2A (see review [65]). PKCα is reciprocally controlled by the activity of the phosphatase, which limits the catalytic action of the kinase by dephosphorylation [66–70].

PP2A itself is a heterotrimeric enzyme comprising one each of two variant catalytic and structural sub-units together with one (of a family of about twenty) modulatory/targeting sub-unit. The nature of the latter determines the targeting and substrate specificity of the assembled enzyme complex. C-terminal carboxymethylation of the catalytic sub-unit at Leu^309^ activates PP2A probably by facilitating the formation of trimeric complexes [71–73]. At the membrane, the enzyme may be phosphorylated on Tyr^307^ by receptor or other tyrosine kinase action [74] that may inhibit the phosphatase. However, the significance of these post-translational modifications to enzyme activity *in vitro* and *in vivo* is not yet entirely clear [65].

The degree of Anx-A1 phosphorylation, and hence the amount exported, will therefore depend upon the net catalytic activity of the PKCα–PP2A complex which, in turn will result from the reciprocal interactions between the two enzymes. The cumulative evidence from biochemical, immunocytochemical and imaging studies presented here all support the notion that whilst the cromoglycate-like drugs alone have only weak activity they are able greatly to accelerate the phosphorylation and release of Anx-A1 when this is primed (in this case) by GCs, which promote PKC activation and some degree of Anx-A1 phosphorylation and membrane localisation. Our analysis points to a direct inhibitory action of these drugs on PP2A enzyme activity as the most likely explanation for this synergism although we cannot entirely rule out the participation of PP1, another phosphatase, or the possibility that which ever phosphatase is involved, it acts directly to dephosphorylate Anx-A1 itself rather than PKC. Supporting the notion that PP2A is the actual target however is our observation that the PP2A inhibitor okadaic acid [75], which has been shown in many systems to potentiate the effects of PKC mediated events, mimics the effect of the cromoglycate-like drugs and promoted GC-stimulated Anx-A1 export in our system.

Experiments using anti-Anx-A1 neutralising antibodies [4,7,9,76–78], anti-sense RNA [79,80] or transgenic animals [20,81–84] which over- or under-express the Anx-A1 gene, have conclusively demonstrated that this protein is a key mediator of GC action in the innate as well as the adaptive [85,86] immune system as well as in other aspects of physiology [87,88] and cell biology [89–91].

Although there are inflammatory models in which Anx-A1 is not efficacious [5], our observation that cromoglycate drugs promote Anx-A1 externalisation may explain several of their disparate actions including their ability to inhibit leukocyte activation [92], ‘priming’ or migration [93,94], mediator action [95], macrophage activation [96], tachykinin action [97], eicosanoid [41,42] and cytokine release [98] as well as adhesion molecule expression [99]. We can only conjecture as to whether our putative mechanism of action accounts for the ability of the cromoglycate-like drugs to inhibit the release of mast cell histamine but it is certainly true that Anx-A1 has potent inhibitory effects on mast cells [17] and that these cells are a prime site for synthesis of this protein [100].

Whilst our hypothesis is novel, there have been several previous attempts to link cromolyn action to activation of signalling pathways and modification of potential down-stream molecular targets. Treatment of mast cells with cromoglycate results in the phosphorylation of (at least) four intracellular protein substrates including the erythrocyte band 4.1 group protein moesin [101,102] and there have been scattered reports of an interaction between cromoglycate and PKC stretching back over some years (e.g. Refs. [103–105]). However, most researchers seem to have considered that these drugs inhibit, rather than stimulate, this enzyme, although differing experimental protocols and crucially, timing, obscure clear interpretation of this issue. Congruent with our findings there is a report that some biological effects of methylprednisolone can be augmented using okadaic acid, an inhibitor of PP2A [106].

There have also been previous reports [107,108] of a link between the cromoglycate drugs and the annexin system in that these drugs have an affinity for some S100 proteins that are intracellular binding partners for members of the annexin family of proteins. It is not clear if, or how these data relate to the current findings.

One practical implication of this work arises from the observation that GCs and cromoglycate-like drugs exhibit a strong synergistic action that might be applicable to anti-inflammatory therapy. The notion that these drugs can inhibit PP2A may also suggest other uses for these agents in other conditions where PKC activation plays a significant role.

## Conflict of interest

The authors have no conflicting financial interests.
